# Combination of volume and perfusion parameters reveals different types of grey matter changes in schizophrenia

**DOI:** 10.1038/s41598-017-00352-z

**Published:** 2017-03-27

**Authors:** Lixue Xu, Wen Qin, Chuanjun Zhuo, Huaigui Liu, Jiajia Zhu, Chunshui Yu

**Affiliations:** 1 Department of Radiology and Tianjin Key Laboratory of Functional Imaging, Tianjin Medical University General Hospital, Tianjin, 300052 China; 2 Tianjin Anding Hospital (Tianjin Mental Health Center), Tianjin, 300222 China; 3 Tianjin Anning Hospital, Tianjin, 300300 China

## Abstract

Diverse brain structural and functional changes have been reported in schizophrenia. Identifying different types of brain changes may help to understand the neural mechanisms and to develop reliable biomarkers in schizophrenia. We aimed to categorize different grey matter changes in schizophrenia based on grey matter volume (GMV) and cerebral blood flow (CBF). Structural and perfusion magnetic resonance imaging data were acquired in 100 schizophrenia patients and 95 healthy comparison subjects. Voxel-based GMV comparison was used to show structural changes, CBF analysis was used to demonstrate functional changes. We identified three types of grey matter changes in schizophrenia: structural and functional impairments in the anterior cingulate cortex and insular cortex, displaying reduction in both GMV and CBF; structural impairment with preserved function in the frontal and temporal cortices, demonstrating decreased GMV with normal CBF; pure functional abnormality in the anterior cingulate cortex and lateral prefrontal cortex and putamen, showing altered CBF with normal GMV. By combination of GMV and CBF, we identified three types of grey matter changes in schizophrenia. These findings may help to understand the complex manifestations and to develop reliable biomarkers in schizophrenia.

## Introduction

Schizophrenia is a neuropsychiatric disorder with complicated structural and functional changes, resulting in diverse clinical manifestations. The grey matter volume (GMV) is the most frequently used and reliable imaging index to characterize brain structural changes. GMV reduction has been considered as the most prominent structural changes in schizophrenia^1^. The reduced GMV has been found in multiple brain regions^2–4^ and in various schizophrenia related subpopulations^5–7^. The cerebral blood flow (CBF) is a reliable imaging index to characterize brain functional changes. The CBF is closely coupled with glucose utilization and oxygen consumption, thus reflects the local neuronal activity^8^. Both increased and decreased regional CBF have been observed in schizophrenia^9, 10^.

Identifying different types of brain changes may improve our understanding of the neural mechanisms and facilitate the development of reliable biomarkers for schizophrenia. The combination of brain structural and functional changes may help to identify several types of meaningful regional brain alterations in schizophrenia. Recently, one study have applied structure and function parameters to distinguish schizophrenia patients from healthy comparison controls, and found that combination of structural and functional magnetic resonance imaging (MRI) data outperformed single MRI modality^11^, which implied that integration of structural and functional analysis could be more effective to describe the cerebral aberration of schizophrenia. However, no prior attempt has been done to combine volume and perfusion parameters to categorize brain change patterns in schizophrenia.

We assumed that there are at least three types of brain changes in schizophrenia. If brain regions exhibit both structural and functional impairments, these regions would probably be the primary causes for clinical symptoms. If brain regions show structural impairments with normal function or functional compensation, effective treatments may prevent these impairments from progressing into functional decompensation. And if structurally normal brain regions demonstrate functional abnormalities, pointed treatments may recover these abnormal functions.

In this study, the voxel-based GMV comparisons between patients and controls were used to identify structurally impaired brain regions in schizophrenia. In brain regions with GMV reduction, the voxel-based CBF comparisons were performed to further identify regions with and without CBF reduction. The similar CBF analysis was performed in brain regions without GMV reduction to detect pure functional abnormality in schizophrenia.

## Methods and Materials

### Participants

A total of 100 schizophrenia patients and 95 healthy comparison subjects were recruited for this study. Diagnoses for patients were confirmed using the Structured Clinical Interview for DSM-IV. The inclusion criteria included age (18–55 years), Chinese Han population, and right-handedness. The exclusion criteria included MRI contraindications, poor image quality, or a history of a systemic medical illness, CNS disorder or substance abuse. Additional exclusion criteria for the healthy comparison subjects included a history of any Axis I or II disorders, diagnosis of a psychotic disorder or a first-degree relative with a psychotic disorder. The clinical symptoms of psychosis were quantified with the Positive and Negative Syndrome Scale (PANSS)^12^. This study was approved by the Medical Research Ethics Committee at Tianjin Medical University General Hospital, and after complete description of the study, written informed consent was obtained from each participant. All methods were performed in accordance with the relevant guidelines and regulations.

### Image data acquisition

MRI was performed using a 3.0-Tesla MR system (Discovery MR750, General Electric, Milwaukee, WI, USA). Tight but comfortable foam padding was used to minimize head motion, and earplugs were used to reduce scanner noise. Sagittal 3D T1-weighted images were acquired by a brain volume sequence with the following parameters: repetition time (TR) = 8.2 ms; echo time (TE) = 3.2 ms; inversion time = 450 ms; flip angle (FA) = 12°; field of view (FOV) = 256 mm × 256 mm; matrix = 256 × 256; slice thickness = 1 mm, no gap; and 188 sagittal slices. The resting-state perfusion imaging was performed using a three-dimensional pseudo-continuous arterial spin labeling imaging (pcASL) sequence with a 3D fast spin-echo acquisition and background suppression: TR = 4886 ms; TE = 10.5 ms; post-label delay = 2025 ms; spiral in readout of eight arms with 512 sample points; FA = 111°; FOV = 240 mm × 240 mm; matrix = 128 × 128; slice thickness = 4 mm, no gap; 40 axial slices; and number of excitation = 3. During the ASL scans, all subjects were instructed to keep their eyes closed, to relax and move as little as possible, to think of nothing in particular, and to not fall asleep. After MRI scans, we ask subjects whether they have done as instructed.

### GMV calculation

The GMV of each voxel was calculated using Statistical Parametric Mapping software (SPM8; http://www.fil.ion.ucl.ac.uk/spm/software/spm8/). The structural MR images were segmented into grey matter (GM), white matter and cerebrospinal fluid using the unified segmentation model. After an initial affine registration into Montreal Neurological Institute (MNI) space, GM concentration images were nonlinearly warped using diffeomorphic anatomical registration through the exponentiated lie algebra (DARTEL) technique^13^ and were resampled to 2-mm cubic voxels. The GMV of each voxel was obtained by multiplying the GM concentration map by the non-linear determinants derived from the spatial normalization step^14^. Finally, GMV maps were smoothed with a Gaussian kernel with a 6 mm × 6 mm × 6 mm full-width at half maximum (FWHM). Two researchers performed the analyses independently. Then we check the consistency of results between the two researchers.

### CBF calculation

The ASL difference images were calculated after the subtraction of the label images from the control images. The CBF maps were derived from the ASL difference images. The detailed calculation procedures have been described in a previous study^15^. SPM8 software was used to coregister the CBF images of the 95 healthy comparison subjects to a PET-perfusion template in the MNI space using non-linear transformation. The CBF template was defined as the mean CBF image of the 95 healthy comparison subjects. The CBF images of all participants, including patients and controls, were subsequently coregistered to the MNI-standard CBF template and were resampled to 2-mm cubic voxels. The CBF of each voxel was normalized by dividing the mean CBF of the whole brain^16, 17^. Then the resulting CBF maps were spatially smoothed with a Gaussian kernel of 6 mm × 6 mm × 6 mm FWHM. Two researchers performed the analyses independently. Then we check the consistency of results between the two researchers.

### Voxel-based group comparisons

The coregistered GMV and CBF maps with a voxel size of 2 mm × 2 mm × 2 mm were used for voxel-based group comparisons, which were performed using a two-sample t-test, controlling for the effects of age and sex. Multiple comparisons were corrected using a family-wise error (FWE) method (p < 0.05, two-tailed, cluster size >100 voxels). Several steps were used to identify different types of grey matter changes in schizophrenia. First, GMV comparisons between groups were used to differentiate GM regions with and without GMV changes in schizophrenia. Second, group comparisons in CBF were performed in brain regions with GMV reduction in schizophrenia to identify the functional changes in these structurally impaired regions. Third, group comparisons in CBF were performed in brain regions without GMV changes in schizophrenia to identify the functional changes in these structurally normal regions.

Decreased GMV was found in several brain regions in our study, to investigate the potential effect of GMV on CBF, we performed an additional voxel-based group analysis. A general linear model with GMV as an additional covariate was used to recalculate CBF differences in brain regions with CBF changes (p < 0.05, Bonforroni corrected).

### Correlation analyses

To test correlations between different types of brain changes and clinical variables, we drew a sphere with a radius of 6 mm centred at the centre of gravity of each region, and then we extracted imaging measurements of the sphere and calculated Spearman’s correlation coefficients between the imaging measurements of the sphere and the clinical parameters (i.e., PANSS score, duration of illness, and antipsychotic dosage). A value of uncorrected p < 0.05 was considered significant.

### Resting-state functional connectivity calculation

Abnormal GMV and CBF were found in bilateral anterior cingulate cortex (ACC) in our study. To confirm the relationship between ACC and default mode network as well as salience network, we applied a functional connectivity analysis, we regarded each ACC as a region of interest (ROI) and calculate functional connectivity map of each ACC in healthy control subjects (Please see the detailed method in the method section of Supplement 1).

## Results

### Demographic and clinical characteristics of subjects

We finally included 100 schizophrenia patients (58 males; age: 34.1 ± 8.2 years) and 95 healthy comparison subjects (44 males; age: 33.3 ± 10.4 years). The demographic and clinical characteristics of these subjects are summarized in Table 1. There were no significant group differences in sex (χ^*2*^ = 2.666, p = 0.103) and age (t = −0.532, p = 0.595). To further exclude the effects of sex and age on our results, we considered these two variables as covariates of no interest throughout the GMV and CBF analyses. Ninety-two patients were receiving atypical antipsychotics at the time of the MRI examinations; eight patients had never received any medications.

**Table 1:** Demographic and clinical characteristics of participants.

Characteristics	Healthy comparison subjects (n = 95)			Schizophrenia patients (n = 100)			Statistics	
	Mean	SD	Range	Mean	SD	Range	t/χ^2^	p
Age (years)	33.3	10.4	20–57	34.1	8.2	20–59	t = −0.532	0.595
Sex	M44	F51	—	M58	F42	—	χ^2^ = 2.666	0.103
Duration of illness (months)	—	—	—	126.3	97.7	0–468	—	—
Positive and negative syndrome scale score								
Positive subscore	—	—	—	16.9	7.8	—	—	—
Negative subscore	—	—	—	20.2	9.0	—	—	—
General subscore	—	—	—	71.2	22.8	—	—	—
Current antipsychotic dosage (chlorpromazine equivalents)^47^ (mg/d)	—	—	—	451.8	342.3	0–1800	—	—

### GMV differences between groups

Compared with healthy comparison subjects, schizophrenia patients showed significantly reduced GMV in the bilateral insular cortex, ACC and thalamus and the left middle temporal gyrus (MTG) (p < 0.05, FWE correction) (Fig. S1 in the Supplement 1). No significantly increased GMV was observed in schizophrenia patients.

### Brain regions with reduced GMV and CBF in schizophrenia

**Figure 1:**
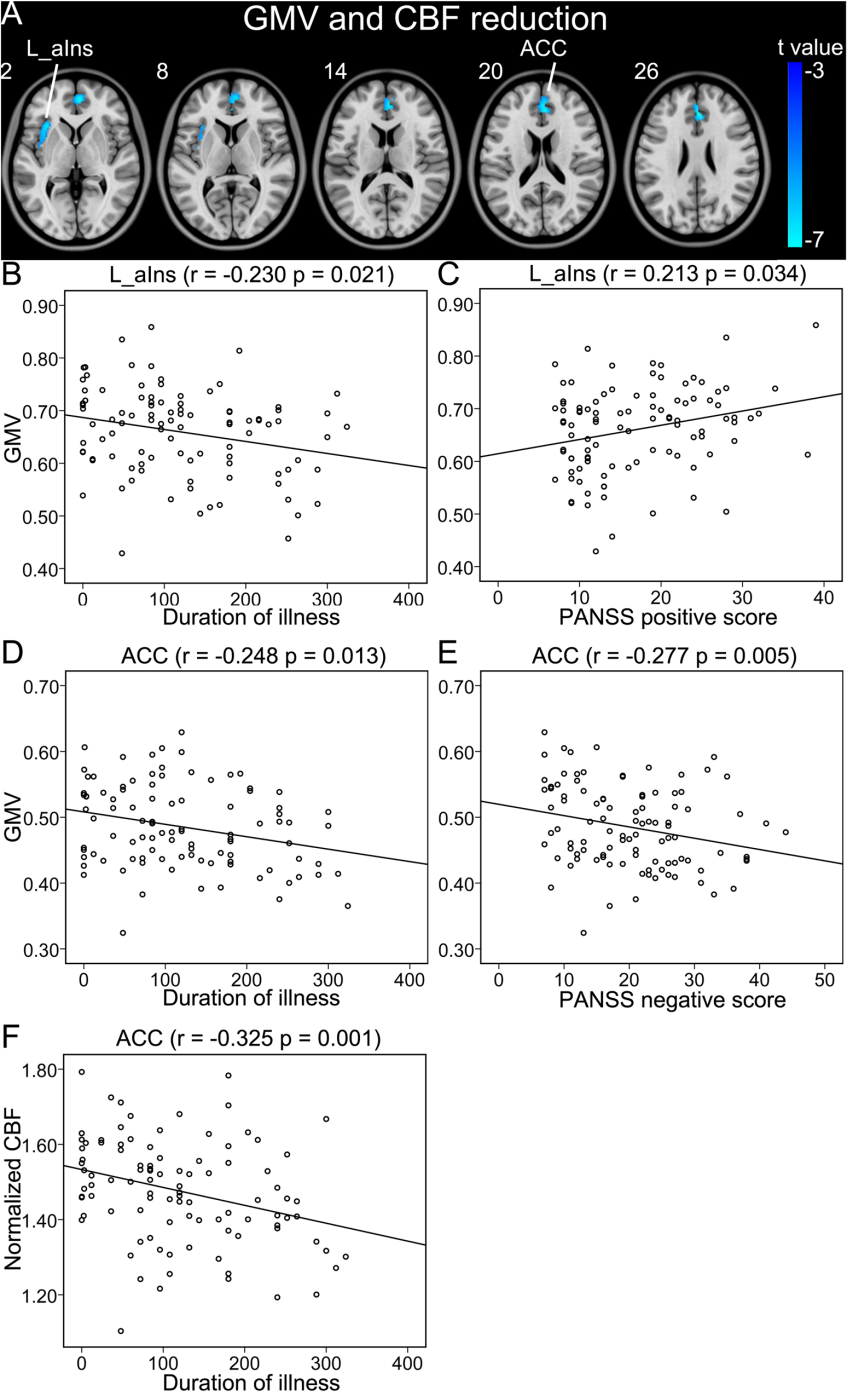
Brain regions with both reduced GMV and CBF in schizophrenia patients (**A**). (**B**–**F**) Shows correlations between GMV and CBF of these regions and clinical parameters in schizophrenia patients. Abbreviations: CBF, cerebral blood flow; GMV, grey matter volume; L, left; and R, right; ACC, anterior cingulate-cortex; aIns, anterior insular cortex. The r value represents the Spearman’s correlation coefficients.

Within brain regions with significant GMV reduction in schizophrenia, we also found significant CBF reduction in the ACC and the left anterior insular cortex (p < 0.05, FWE correction) (Table 2, Fig. 1A). The GMV (r = −0.248, p = 0.013) and CBF (r = −0.325, p = 0.001) of the ACC and the GMV of the left anterior insular cortex (r = −0.230, p = 0.021) were negatively correlated with the duration of illness (Fig. 1B,D,F). The GMV of the ACC was negatively correlated with PANSS negative score (r = −0.277, p = 0.005) and the GMV of the left anterior insular cortex was positively correlated with the PANSS positive score (r = 0.213, p = 0.034) in schizophrenia patients (Fig. 1C,E).

**Table 2:** GMV, CBF and CBF/GMV ratio changes in schizophrenia patients.

Brain regions*	MNI coordinates (x, y, z)	Peak t value	Cluster size (Voxels)**
GMV and CBF reduction			
Anterior cingulate cortex	2, 36, 24	−7.23	699
Left anterior insular cortex	−34, 22, 2	−6.38	248
GMV reduction and normal CBF			
Right insular cortex	33, 22, −4	−7.56	1596
Left medial prefrontal cortex	−8, 50, 14	−7.12	1118
Left amygdala	−20, 2, −18	−6.99	420
Left superior temporal gyrus	−52, 0, −2	−6.83	508
Right posterior mid-cingulate cortex	8, 18, 36	−6.57	212
Left posterior mid-cingulate cortex	−8, 22, 34	−6.20	138
Left middle temporal gyrus	−60, −36, 2	−6.02	188
Thalamus	0, −6, 4	−5.57	203
Reduced CBF and normal GMV			
Right anterior cingulate cortex	4, 36, 24	−7.14	768
Left lateral prefrontal cortex	−30, 60, 2	−6.62	418
Increased CBF and normal GMV			
Left putamen	−30, −10, −2	6.38	125
Right putamen	32, −2, 2	6.05	236

*Multiple comparisons are corrected using a family-wise error method (p < 0.05, cluster size >100 voxels).

**Voxel size is 2 × 2 × 2 mm^3^.

### Brain regions with reduced GMV and normal CBF in schizophrenia

**Figure 2:**
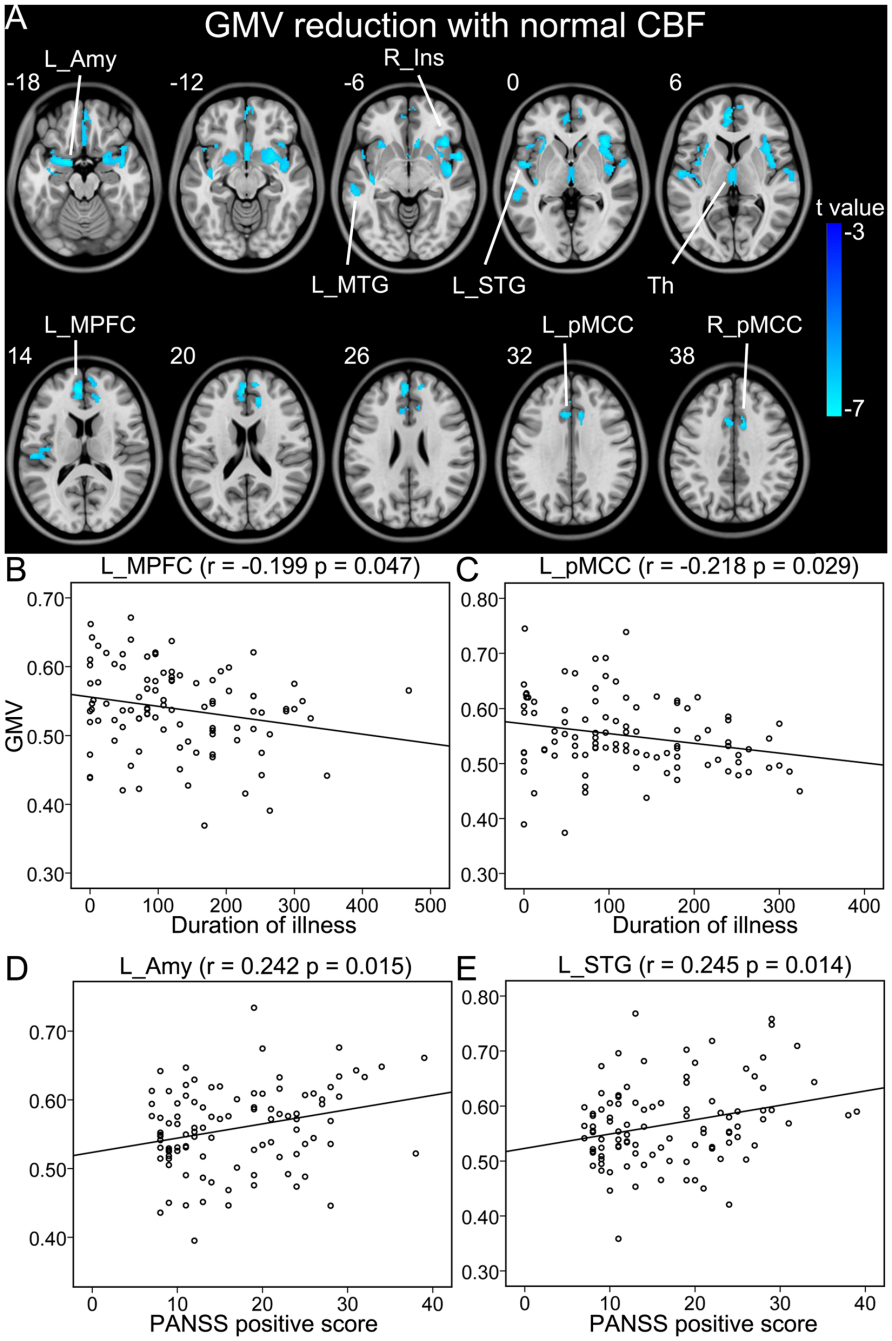
Brain regions with reduced GMV and normal CBF in schizophrenia patients (**A**). (**B**–**E**) Shows correlations between GMV and CBF of these regions and clinical parameters in schizophrenia patients. Abbreviations: CBF, cerebral blood flow; GMV, grey matter volume; L, left; and R, right; Amy, amygdala; Ins, insular cortex; MPFC, medial prefrontal cortex; MTG, middle temporal gyrus; pMCC, posterior middle cingulate-cortex; STG, superior temporal gyrus. The r value represents the Spearman’s correlation coefficients.

Within brain regions with significant GMV reduction in schizophrenia, normal CBF was observed in the right insular cortex, the left MPFC, amygdala, superior temporal gyrus (STG) and MTG, and the bilateral thalami and posterior MCC (pMCC) (Table 2, Fig. 2A). The GMVs of the left pMCC (r = −0.218, p = 0.029) and MPFC (r = −0.199, p = 0.047) were negatively correlated with the duration of illness. The GMVs of the left amygdala (r = 0.242, p = 0.015) and STG (r = 0.245, p = 0.014) were positively correlated with the PANSS positive score in schizophrenia patients (Fig. 2B–E).

### Brain regions with normal GMV and abnormal CBF in schizophrenia

**Figure 3:**
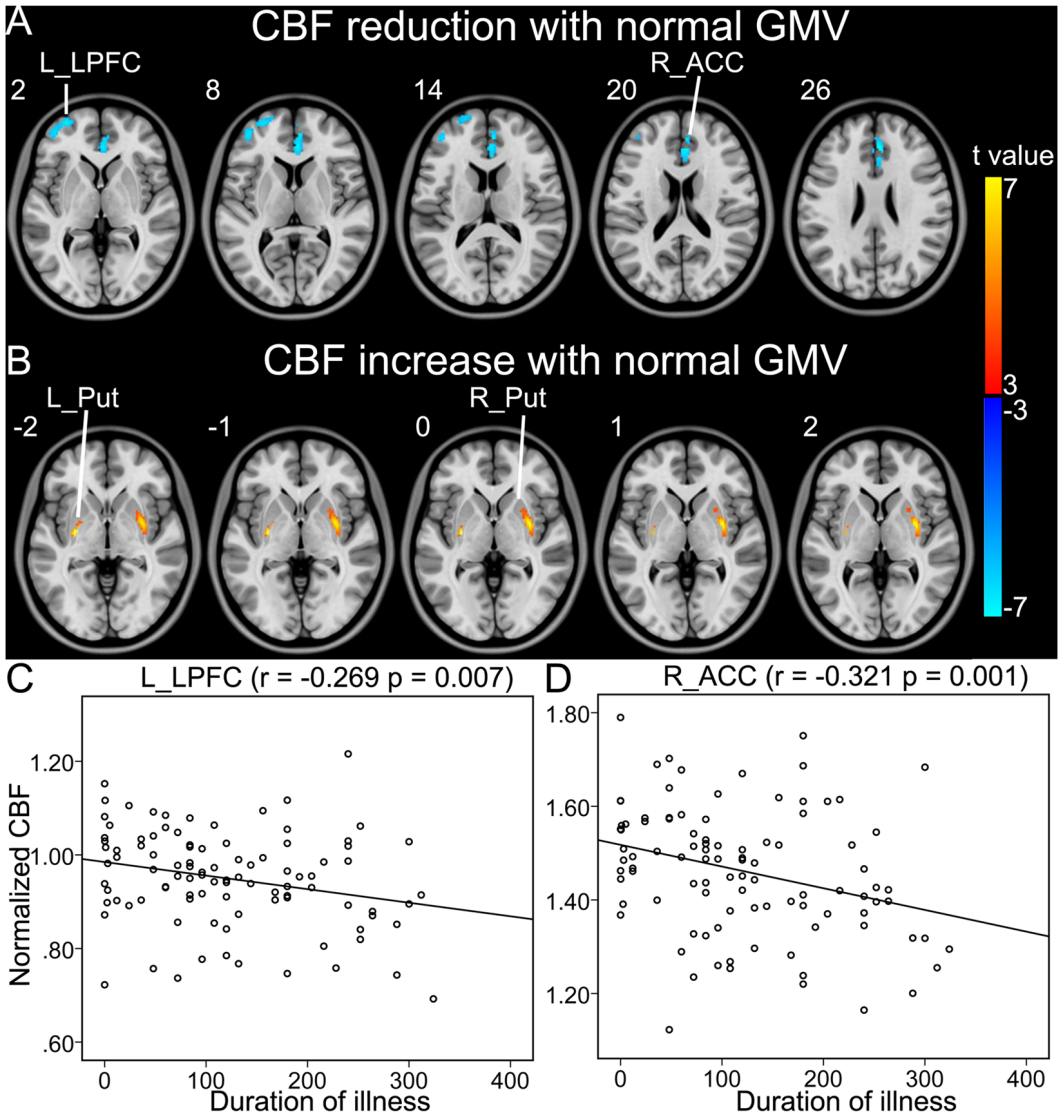
Brain regions with normal GMV and abnormal CBF in schizophrenia patients. (**A**) Shows brain regions with normal GMV and reduced CBF in patients. (**B**) Shows brain regions with normal GMV and increased CBF in patients. (**C**,**D**) Show correlations between CBF of these regions and clinical parameters in schizophrenia patients. Abbreviations: CBF, cerebral blood flow; GMV, grey matter volume; L, left; and R, right; ACC, anterior cingulate-cortex; LPFC, lateral prefrontal cortex; Put, putamen. The r value represents the Spearman’s correlation coefficients.

In brain regions without significant GMV changes, schizophrenia patients showed significantly reduced CBF in the right ACC and the left lateral prefrontal cortex (LPFC) and significantly increased CBF in the bilateral putamen than healthy comparison subjects (Table 2, Fig. 3A,B). The CBF values of the LPFC (r = −0.269, p = 0.007) and ACC (r = −0.321, p = 0.001) were negatively correlated with the duration of illness in schizophrenia patients (Fig. 3C,D).

### Potential effect of GMV on CBF

We found that CBF alteration remained significant after GMV correction (p < 0.05 with Bonforroni corrected) (Fig. S2 in Supplement 1). Thus, CBF alterations are not likely caused by GMV alterations.

### Potential effect of smoothing kernels on GMV and CBF

In addition, we choose a Gaussian kernel of 4 FWHM, 8 FWHM and 10 FWHM to validate whether brain regions showed significantly abnormal GMV and/or CBF in our study were influenced by the Gaussian kernel. We extracted the signal of GMV or CBF in each significant brain region and adopted a general linear model to compare group differences with sex and age as covariates. The results are listed in Tables S1 and S2 in Supplement 1. We found that the results were still significant when we used other smoothing kernels.

## Discussion

Based on GMV and CBF changes, we identified three types of grey matter changes in schizophrenia: (1) both GMV and CBF reduction in the ACC and anterior insular cortex; (2) GMV reduction with normal CBF in the insular cortex, MPFC, pMCC, STG, MTG, thalamus and amygdala; and (3) CBF reduction with normal GMV in the ACC and LPFC as well as CBF increase with normal GMV in the putamen.

Both GMV and CBF reduction were found in the anterior insular cortex and ACC in schizophrenia patients, indicating both structural and functional impairments in these regions. The anterior insular cortex is an important node of the salience network (SN), which serves to identify and assess salient events, and to initiate appropriate signals to control human behaviors via switching between central executive network and default mode network (DMN)^18^. The structural and functional impairments in the anterior insular cortex is consistent with previous studies that reporting reduced GMV^19^ and white matter integrity^20^, and abnormal task-evoked activation^21^ in this region in schizophrenia. The impairment of the anterior insular cortex may be an important cause for the dysfunction of the SN^16, 22, 23^, which resulting in aberrant salience attribution and contributing to the formation of schizophrenic core symptoms^24, 25^. This is confirmed by our correlation finding between the GMV of the anterior insular cortex and PANSS positive score. The resting-state functional connectivity analysis revealed that the impaired ACC region was associated with both the SN and DMN (Fig. S3 in the Supplement 1), suggesting that it is the connected hub of the two networks. Thus, the impairment of the ACC region destructs not only these two networks themselves but also their connections. The DMN plays a role in social cognition and emotional regulation^26, 27^, dysfunction of which is frequently reported in schizophrenia^28, 29^. The structural and functional impairments in the ACC have been found in both schizophrenia patients and their first-degree relatives^4, 30, 31^. In consistent with previous studies^32^, we found that the structural impairment of the ACC was correlated with clinical symptoms. Consequently, the ACC and the anterior insular cortex, the critical nodes of the SN and DMN, are the most impaired regions in schizophrenia, which may underlie the core dysfunctions in schizophrenia^33^. The structural and functional features of these regions may be used as objective biomarkers for schizophrenia.

Several brain regions with reduced GMV showed normal CBF in schizophrenia. The MPFC and amygdala are components of the corticolimbic circuit which is responsible for affective arousal and regulation. In consistent with a previous study^34^, we found GMV reduction in the MPFC and amygdada. Moreover, we found a positive correlation between the GMV of the amygdala and PANSS positive score, supporting the impairment of this circuit contributing to the affective symptoms in schizophrenia^35^. The pMCC involves in sensorimotor integration and shows functional disconnection in schizophrenia^36, 37^, the GMV reduction in the pMCC provides evidence for the structural impairment of this region in schizophrenia. GMV reduction was also found in the thalamus, which has been related to cognitive deficits in schizophrenia^38^. In agreement with previous studies^5, 39^, we found GMV reduction in the MTG and STG. These regions involve in auditory and language processing, the impairments of which are associated with core symptoms of schizophrenia, such as auditory hallucination^40^. This finding is further supported by the correlation between the GMV of the left STG and PANSS positive score in our study. The normal CBF in these structurally impaired regions provides evidence of their asynchronization between function and structure impairment, suggesting that effective treatments may prevent these regions from progressing into the stage of functional decompensation.

Among brain regions with normal GMV, several regions displayed abnormal CBF in schizophrenia. As nodes of the SN, the LPFC and ACC showed reduced CBF in schizophrenia, which may also contribute to the dysfunction of the SN. We also found increased CBF in the putamen in schizophrenia, which is consistent with previous perfusion studies^9, 41^. The putamen contains rich dopaminergic neurons and is involved in motor, cognition and emotional processing and abnormal activation of putamen was associated with hallucination in schizophrenia^42^. The hyper-perfusion in the caudate nucleus has been attributed to antipsychotic treatment^43^; however, the lack of correlation between the CBF of the putamen and antipsychotic dosage in our study and previous studies^41, 44^ suggests that whether the striatum is a treatment biomarker needs further validation. The functional abnormalities in structurally normal brain regions may be important targets for antipsychotic treatments, which have the potential to recover these abnormal functions.

In consistent with previous studies reporting progressively structural and functional impairments in schizophrenia^45^, we also found significant negative correlations between the regional GMV and CBF and the duration of illness in schizophrenia patients. These findings suggest that structural and functional integrity may be progressively damaged following disease course of schizophrenia. Therefore, earlier intervention is very important for delaying or preventing the structural and functional impairment in schizophrenia.

There are several limitations that should be mentioned in this study. First, most patients were at the chronic stage of schizophrenia, which prevents us from elucidating the types of brain changes at onset of the disease. This information may help to differentiate brain changes resulted from the developmental or the neurodegenerative mechanism. Second, ninety-two out of 100 schizophrenia patients received antipsychotic treatment during MR scans, we couldn’t rule out the possible effect of medication on our findings because increased CBF has been reported in the putamen after the administration of antipsychotic medication^46^. Although we didn’t find any significant correlations between imaging parameters and antipsychotic dosage, future studies with medication-naïve, first-episode schizophrenia patients are required to eliminate the possible medication effects in this study. Third, the correlations between imaging measurements and PANSS score presented in our study were not soundly significant, which made our interpretation less convictive.

In summary, we combined the GMV and CBF to identify brain change patterns in schizophrenia. We identified three types of brain changes: structural and functional impairments; structural impairment with preserved function; and pure functional abnormalities. These findings may improve our understanding of the neural mechanisms and facilitate the development of reliable biomarkers for schizophrenia.

## Electronic supplementary material

Supplement 1
