# Supplementary material for: Combination of volume and perfusion parameters reveals different types of grey matter changes in schizophrenia
Source: Sci Rep. 2017 Mar 27;7:435. doi: 10.1038/s41598-017-00352-z (PMC5428274; doi:10.1038/s41598-017-00352-z)
Supplement: Supplementary file 1 — Supplement 1 [file 41598_2017_352_MOESM1_ESM.doc]

**Full Title:**

**Combination of volume and perfusion parameters reveals different types of grey matter changes in schizophrenia**

**Running Title:**

**Different types of grey matter changes in schizophrenia**

**Lixue Xu ^1, 4^, Wen Qin ^1, 4^, Chuanjun Zhuo ^2, 3, 4^ , Huaigui Liu ^1^, Jiajia Zhu ^1^, and Chunshui Yu*^, 1^**

^1^ Department of Radiology and Tianjin Key Laboratory of Functional Imaging, Tianjin Medical University General Hospital, Tianjin 300052, China

^2^ Tianjin Anding Hospital (Tianjin Mental Health Center), Tianjin City 300222, China

^3^ Tianjin Anning Hospital, Tianjin City 300300, China

^4^ These authors contributed equally to the article.

***Address correspondence to:** Dr. Chunshui Yu.

Department of Radiology, Tianjin Medical University General Hospital, No. 154, Anshan Road, Heping District, Tianjin 300052, China.

E-mail: [chunshuiyu@vip.163.com](mailto:chunshuiyu@vip.163.com)

**Supplementary Methods**

**Image data acquisition**

A gradient-echo single-shot echo planar image sequence was performed using the following parameters: TR/TE = 2000/45 ms; FOV = 220 mm × 220 mm; matrix = 64 × 64; FA = 90°; slice thickness = 4 mm; gap = 0.5 mm; 32 interleaved transverse slices; 180 volumes. All slices were parallel to the AC-PC line. No particular adjustments for shimming or gradient moment correction were applied.

**Functional magnetic resonance imaging (fMRI) preprocessing**

We only focued on the healthy comparison subjects to avoid potential confounders associated with antipsychotic treatment and illness duration ^1^. Therefore, functional images of 95 healthy comparison subjects were obtained. The first 10 volumes for each subject were discarded to allow the signal to reach equilibrium and the participants to adapt to the scanning noise. The remaining volumes were then corrected for the acquisition time delay between slices. All subjects’ fMRI data were within defined motion thresholds (translational or rotational motion parameters less than 2 mm or 2°). We also calculated framewise displacement (FD), which indexes volume-to-volume changes in head position ^2^. Because recent studies have reported that the signal spike caused by head motion significantly contaminates final resting-state fMRI results even after regressing out the realignment parameters ^2^, we removed spike volumes if the FD of that specific volume exceeded 0.5. Several nuisance covariates (six motion parameters and the average blood oxygen level-dependent signals of the ventricles, white matter and whole brain) were regressed out from the data. The datasets were band-pass filtered, with a frequency range of 0.01 to 0.08 Hz. Individual structural images were linearly coregistered to the mean functional image; then, the transformed structural images were segmented into grey matter, white matter, and cerebrospinal fluid. The grey matter maps were linearly coregistered to the tissue probability maps in MNI space. Finally the motion-corrected functional volumes were spatially normalized to MNI space using the parameters estimated during linear coregistration. The functional images were resampled into a 3 × 3 × 3 mm^3^ voxel. After normalization, all datasets were smoothed with a Gaussian kernel with a 6 × 6 × 6 mm^3^ full-width at half maximum.

**Resting-state functional connectivity calculation**

Abnormal grey matter volume and cerebral blood flow were found in bilateral anterior cingulate cortex (ACC) in our study. To investigate the functional connectivity maps of bilateral ACC, we regarded each ACC as a region of interest (ROI). For individual datasets, Pearson’s correlation coefficients between the mean time series of each ROI and the time series of each voxel in other parts of the brain grey matter were computed and converted to *z* values using Fisher’s *r*-to-*z* transformation to improve normality. Each individual’s *z* values were then entered into a random-effect one-sample *t*-test in a voxel-wise manner to identify brain regions that showed significant positive correlations with each ACC. Multiple comparisons for these analyses were corrected using a family-wise error (FWE) method (p < 0.05, two-tailed). We only focused on the positive rsFC networks because the biological meaning of negative rsFC networks is a matter of debate ^3,4^.

**Supplementary Figures and Tables**

**
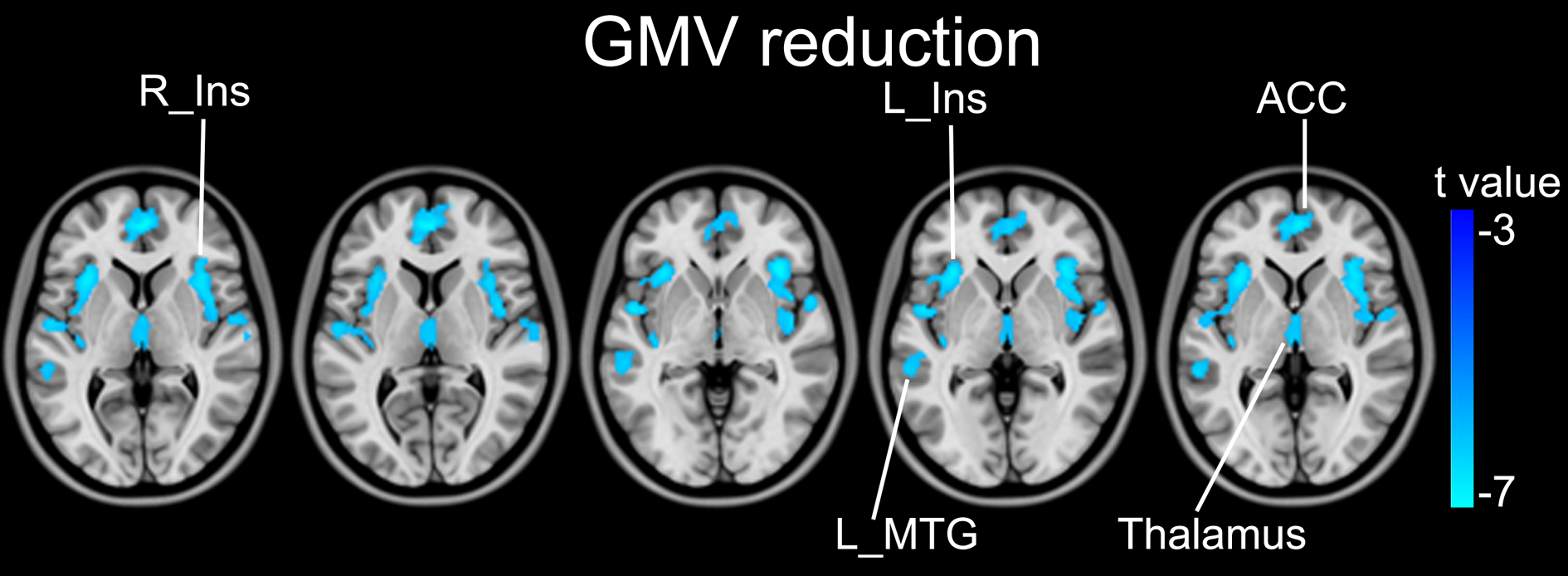
**

**Figure S1.** Brain regions with GMV reduction in schizophrenia patients. Abbreviations: GMV, grey matter volume; L, left; and R, right; ACC, anterior cingulate-cortex; Ins, insular cortex; MTG, middle temporal cortex.

**
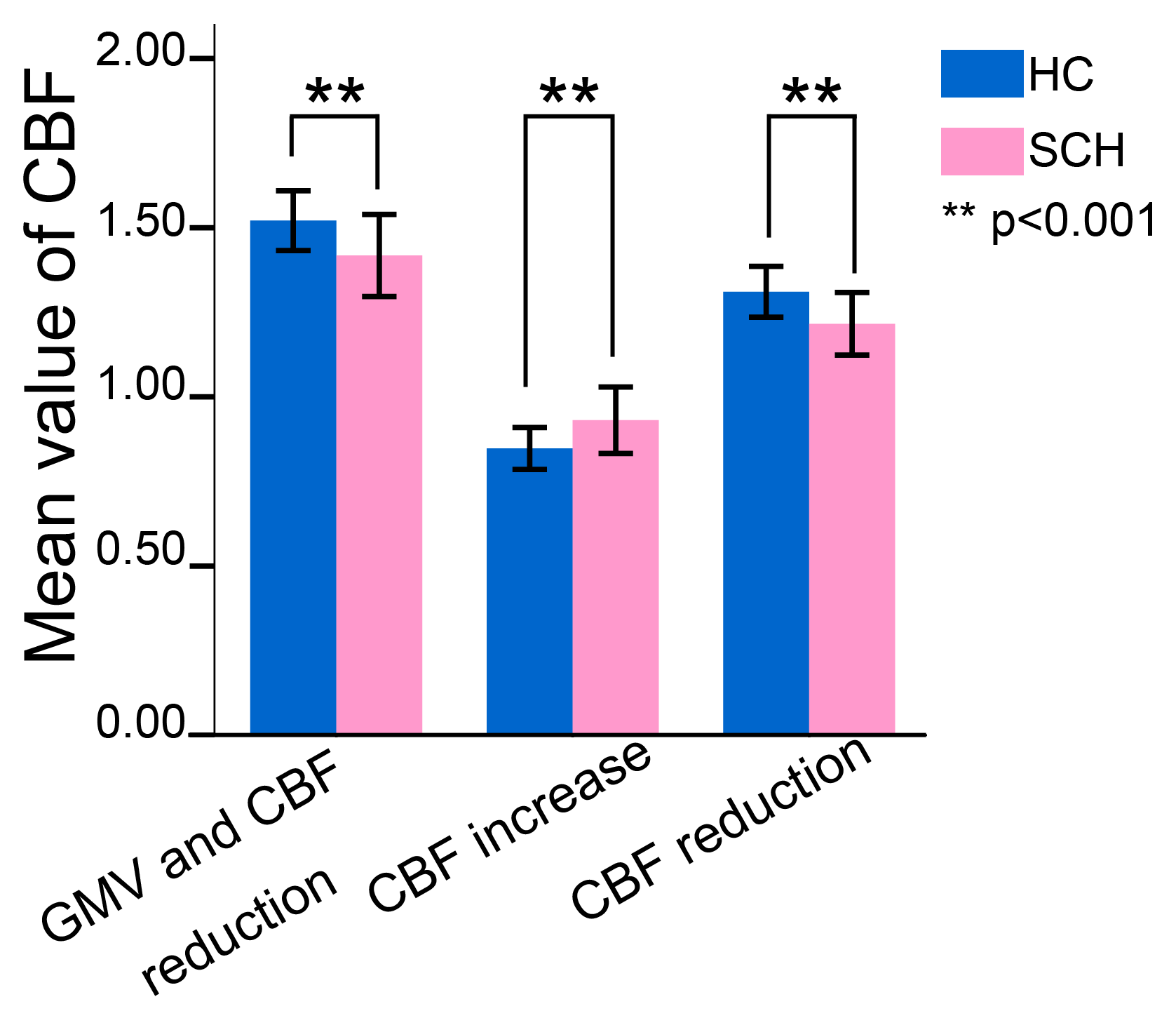
**

**Figure S2.** CBF alterations in schizophrenia after GMV correction. After GMV correction, CBF changes remain significant in brain regions whose CBF altered without GMV correction. Abbreviations: CBF, cerebral blood flow; GMV, grey matter volume; HC, healthy comparison controls; SCH, schizophrenia patients.

**
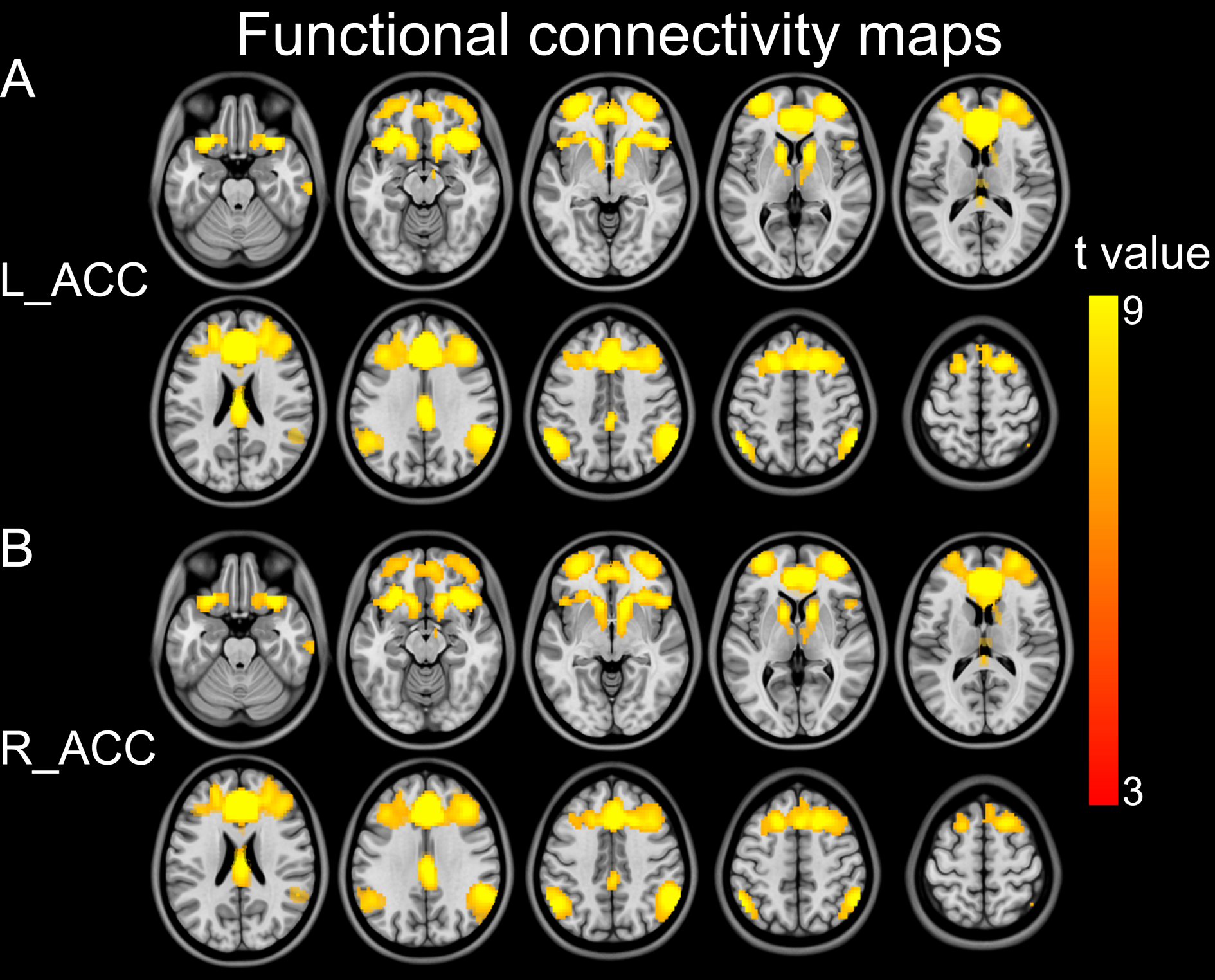
**

**Figure S3:** The resting-state functional connectivity maps of the left anterior cingulate cortex (L_ACC) and the right anterior cingulate cortex (R_ACC) in healthy comparisons. Only the positive resting-state functional connectivity maps of bilateral ACC are depicted. All the images were thresholded at a two-tailed p < 0.05, family-wise error correction, cluster size > 30 voxels.

**Table S1.** Group differences of cerebral blood flow (CBF) using different FWHM for smoothness.

| FWHM | Brain regions with different type of brain changes^a^ | F value | p* value |
| --- | --- | --- | --- |
| 4mm | GMV and CBF reduction | 65.853 | <0.001 |
|  | Increased CBF with normal GMV | 86.413 | <0.001 |
|  | Decreased CBF with normal GMV | 49.474 | <0.001 |
| 8mm | GMV and CBF reduction | 56.474 | <0.001 |
|  | Increased CBF with normal GMV | 71.995 | <0.001 |
|  | Decreased CBF with normal GMV | 42.021 | <0.001 |
| 10mm | GMV and CBF reduction | 50.987 | <0.001 |
|  | Increased CBF with normal GMV | 64.890 | <0.001 |
|  | Decreased CBF with normal GMV | 37.283 | <0.001 |

* The p value remained significant after Bonforroni p<0.05 correction.

^a^ We classified the brain regions with the same type of brain changes as one group, for example, “GMV and CBF reduction” represents the brain regions with both decreased GMV and CBF.

Abbreviations: GMV, grey matter volume. FWHM, full-width at half maximum.

**Table S2.** Group differences of grey matter volume (GMV) using different FWHM for smoothness.

| FWHM | Brain regions with different type of brain changes^a^ | F value | p* value |
| --- | --- | --- | --- |
| 4mm | GMV and CBF reduction | 91.786 | <0.001 |
|  | GMV reduction with normal CBF | 125.152 | <0.001 |
| 8mm | GMV and CBF reduction | 42.021 | <0.001 |
|  | GMV reduction with normal CBF | 76.640 | <0.001 |
| 10mm | GMV and CBF reduction | 71.683 | <0.001 |
|  | GMV reduction with normal CBF | 98.171 | <0.001 |

* The p value remained significant after Bonforroni p<0.05 correction.

^a^ We classified the brain regions with the same type of brain changes as one group, for example, “GMV and CBF reduction” represents the brain regions with both decreased GMV and CBF.

Abbreviations: CBF, cerebral blood flow. FWHM, full-width at half maximum.

**Supplementary references**

1 Hariri, A. R. & Weinberger, D. R. Imaging genomics. *Br Med Bull* **65**, 259-270 (2003).

2 Power, J. D. *et al.* Spurious but systematic correlations in functional connectivity MRI networks arise from subject motion. *Neuroimage* **59**, 2142-2154, doi:10.1016/j.neuroimage.2011.10.018 (2012).

3 Murphy, K. *et al.* The impact of global signal regression on resting state correlations: are anti-correlated networks introduced? *Neuroimage* **44**, 893-905, doi:10.1016/j.neuroimage.2008.09.036 (2009).

4 Weissenbacher, A. *et al.* Correlations and anticorrelations in resting-state functional connectivity MRI: a quantitative comparison of preprocessing strategies. *Neuroimage* **47**, 1408-1416, doi:10.1016/j.neuroimage.2009.05.005 (2009).
